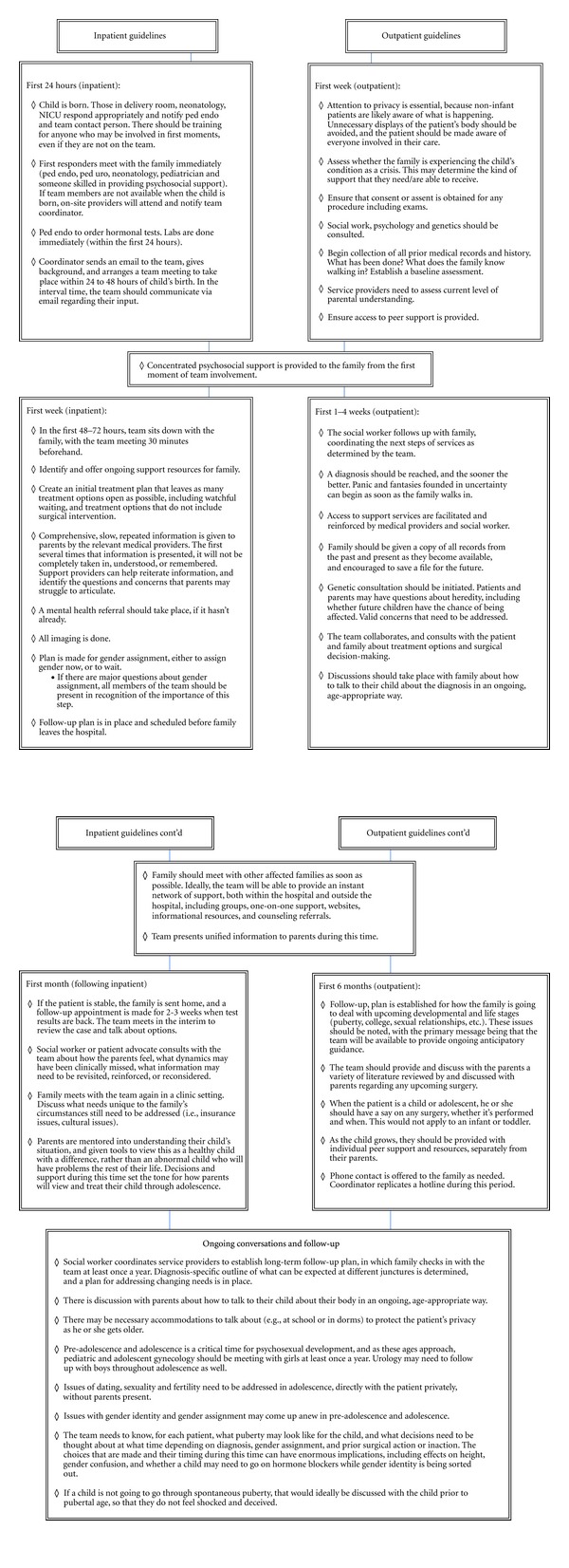# Developing a Multidisciplinary Team for Disorders of Sex Development: Planning, Implementation, and Operation Tools for Care Providers

**DOI:** 10.1155/2012/604135

**Published:** 2012-06-25

**Authors:** Mary Elizabeth Moran, Katrina Karkazis

**Affiliations:** ^1^Department of Sociomedical Sciences, Mailman School of Public Health, Columbia University, 722 West 168th Street, New York, NY 10032, USA; ^2^Center for Biomedical Ethics, Stanford University, 1215 Welch Road, Modular A, Stanford, CA 94305-5417, USA

## Abstract

In the treatment of patients with disorders of sex development (DSD), multidisciplinary teams (MDTs) represent a new standard of care. While DSDs are too complex for care to be delivered effectively without specialized team management, these conditions are often considered to be too rare for their medical management to be a hospital priority. Many specialists involved in DSD care want to create a clinic or team, but there is no available guidance that bridges the gap between a group of like-minded DSD providers who want to improve care and the formation of a functional MDT. This is an important dilemma, and one with serious implications for the future of DSD care. If a network of multidisciplinary DSD teams is to be a reality, those directly involved in DSD care must be given the necessary program planning and team implementation tools. This paper offers a protocol and set of tools to meet this need. We present a 6-step process to team formation, and a sample set of tools that can be used to guide, develop, and evaluate a team throughout the course of its operation.

## 1. Introduction

In the treatment of complex conditions, multidisciplinary teams (MDTs) are increasingly recognized as a best practice in health care delivery [1, 2], with the goal of improving patient satisfaction and outcomes, enhancing the quality of clinical care, and supporting cost containment at the organizational level [3]. In the treatment of children with Disorders of Sex Development (DSD), MDTs represent a new standard of care [4–6], having been shown to provide improved support and care for children and their families [7, 8].

The care required for DSD presents special challenges to providers. Patients and families with DSD require complex medical and psychosocial care, as well as ongoing followup throughout the lifespan [4, 9, 10]. Multidisciplinary care, of which psychosocial and peer support are key components, is recommended as a best practice by experts in DSD management [4, 6, 9]. However, most hospitals do not mandate—or provide administrative, technical, or financial assistance for—the formation of these teams or the inclusion of psychosocial and peer support as standard practice. Consequently, forming a DSD MDT is likely to be a grassroots effort, led by providers, rather than an institutionally sponsored initiative. The burden can lie with the specialists and subspecialists involved in DSD care to create a team or clinic, but specialists likely to participate on a team may not have experience in program planning or team development. This is an important dilemma, and one with serious implications for the future of DSD care. If a network of multidisciplinary DSD teams is to be a reality, those directly involved in DSD care must be given program planning and team development tools. This paper offers a program-planning model and set of tools to help to meet this need.

The model presented in this paper applies established processes to the medical management of DSD. MDTs are routinely employed to manage complex conditions, such as diabetes, cleft palate, and cystic fibrosis [11–14], and program planning and team development processes are established features of organizational development strategies [15, 16]. The model presented in this paper was pilot tested with a group of providers at Lucile Packard Children's Hospital at Stanford University, and the authors have generalized the model to be applicable to any group of providers similarly interested in DSD team development.

This paper acknowledges that, ultimately, the field of DSD care will benefit from being accountable to a standard of excellence that is established, disseminated, and evaluated by a professional association or organization with credibility and authority. Such an organization would define and grant designations of competency and excellence, and many hospitals, in the pursuit of such designations, would likely provide institutional support for teams of providers to achieve and maintain a Center of Excellence (CoE) or specialty team designation. Currently, however, no professional association or organization holds this authority, and neither a set of evidence-based best clinical practices, nor a clear procedure for evaluation and accountability, exist. Providers are therefore faced with the challenge of implementing an interim step in which recommendations for multidisciplinary care are adopted, while future goals of universal treatment and evaluation parameters remain as yet undetermined. This paper presents a model for team formation given the current conditions of DSD research and practice, where long-term outcome data are scarce, ideas about what constitutes ideal patient and family care are diverse, and there is no single agreed-upon definition of successful DSD treatment.

Until there are widely acknowledged, achievable criteria for CoE or authorized specialty DSD clinics, developing teams at all levels of performance is a necessary intermediary step. This is true for three reasons. First, not every potential team will aim to become a CoE but can benefit from multidisciplinary communication and decision-making processes. For a team that does ultimately hope to become a CoE or specialty clinic, this model provides the foundation for unlimited growth and sustainability and will support that end. Second, not every family will have access to CoE or specialty clinics for financial, geographic, structural, or personal reasons. When access to specialized care is not available, teams at local hospitals will bear the responsibility for providing comprehensive DSD care and followup, and a framework for team development will prove valuable. Third, when access to a specialty team is available, there remains a need for skilled, streamlined DSD MDTs in local hospitals, as much of the critical and influential care that a family receives occurs in the very early periods prior to referral.

Team operations will no doubt vary based on the healthcare systems in a given country or culture. The strength of this approach to team formation is that it can be used by, and benefit, any group of medical providers, irrespective of available resources or organizational infrastructure. The model gathers information about what resources and organizational structures are available to the team and then encourages operational design based on those factors. Use of this model will ease decision-making processes, ensure that all team members share equally in information exchange, and ensure that patient families are comprehensively informed and supported.

## 2. Steps to Team Formation and Development

The following section proposes a protocol for DSD MDT development by outlining six steps to team formation: (1) identify and assemble interested team members, (2) assess team capacity, (3) assess resources, (4) interview team members, (5) analyze interview responses, and (6) develop tools and report findings.

### 2.1. Identify and Assemble Interested Team Members

The 2006 Consensus Statement on Management of Intersex Disorders (“Consensus Statement” hereafter) lists an ideal team membership to include “pediatric subspecialists in endocrinology, surgery or urology, psychology/psychiatry, gynecology, genetics, neonatology, and, if available, social work” [4]. Should team representation from all of these areas not be available, an effective team will include, at minimum, a pediatric endocrinologist, a pediatric urologist and/or surgeon, and a psychologist, psychiatrist, and/or social worker who can provide early and ongoing psychosocial care and access to support resources for parents and patients. It is also preferable to have a team member from neonatology or delivery on the team. The early moments in the delivery room when a baby is born with ambiguous genitalia or possible DSD can set the tone for a family's entire experience of the care they receive, and how parents expect the world to engage with their child's condition [17, 18]. Thus, the neonatologist member can help develop and facilitate specialized trainings for all neonatology and delivery personnel in order to ensure that those first moments are handled in a caring, nonstigmatizing way.

The development of a team requires coordination in the planning, implementation, and functioning stages, and a team coordinator should be identified as early as possible. The role of the coordinator can be satisfied either informally by a team member, or formally by assigning coordinating personnel. The coordinator role could be fulfilled by a social worker specially designated for this patient population. A social worker is often the only person routinely talking to the family as well as the team members and can thus provide a consistent link between patients, families, and care providers. An ideal coordinator will be a person trained in the needs of DSD families, who is in a position to readily identify what has been done for each patient, what needs to be done, important markers for followup, and nuances within the patient's family that can inform the path of treatment and support.

Most importantly, the person in the coordination role for the team needs to be committed, driven, and have the team's buy-in. A coordinator will only be effective if he or she has good relationships with team members, is respected, and will be listened to.

### 2.2. Capacity Assessment

 A capacity assessment is an inquiry into what the team is capable of taking on, measuring the realistic capacity of a potential team in terms of time, attention/energy, openness to this new undertaking, and interest in developing professionally in the area of DSD care. Conducting an incisive and accurate capacity assessment is critical to identify areas of limited or excess capacity so that the team can set achievable, meaningful goals. For example, there might be a tremendously enthusiastic team member who can only give one hour every other week to the DSD team. If the team allows that person to take on a large area of responsibility, work-in that area is going to progress slowly despite the team member's enthusiasm because of limitations in time capacity. Alternatively, there may be a team member with greater time capacity who supports the team, but who does not have much self-generated drive. It may be tempting for the team to identify this member as a potential coordinator, but this person will not have the necessary energy and interest for that role. The team's success or failure will ultimately rely on how well the operation plan aligns with the team members' capacity to operate.

A capacity assessment can be done by asking individuals about their time capacity and level of interest in the team, or discussing as a group whether or not the whole team has the time and interest to take on a new project that requires commitment over the long term. Team member interviews come later, so in-depth assessments of each individual's role need not be done at this time. Important questions to answer before finalizing team membership are the following.

- How many hours per week can each individual commit to this team?
- What essential skills need to be present on the team (e.g., strong psychosocial skills), and do any prospective team members actually possess these skills?
- What are some short-term goals that the team can set for the next three months, in order to gauge realistic time commitments and to assess our capacity to follow through?
- Are there prospective team members who are interested in driving the development of the project?

### 2.3. Resource Assessment

Whereas a capacity assessment gauges whether the team has the time and commitment necessary to form and operate as a functional MDT, a resource assessment ascertains the services (such as DSD support groups in the area), programs (such as externship programs with local universities), funding opportunities, support personnel, time, and physical spaces available that can support the team's development and ongoing operation. It is an important assessment to conduct at the earliest stage of team development because the resources available to the team will in many ways shape how (and in some cases whether) the program or clinic will operate. The following are some resource areas to explore.

- Hospital's business department, which can provide guidance on drawing up a business plan for project funding (e.g., program operational costs, continuing education costs via training and conference attendance, funding for social events for children and families);
- internal and external grant opportunities (e.g., a research grant through the hospital, university, or the NIH to study outcomes or assess quality of care);
- internal MDT incentive or facilitation programs (does your hospital incentivize the formation of multidisciplinary teams in any way? Is there already a clinic or division with which you can partner that specializes in complex or rare diagnoses?);
- external consultation resources (e.g., established DSD MDTs that can provide insight and ideas, or recently developed teams and clinics that treat similarly complex and rare conditions such as teams working with children with cystic fibrosis and craniofacial conditions);
- personnel resources available (e.g., staff coordinators in a participating division who can dedicate a percentage of their time to the team, or a staff member who possesses an essential skill not held by current team members);
- team management resources from organizational development literature, such as 360-degree team member performance reviews [19];
- Time and location resources: Is there a time and a venue that can be set aside for a regular outpatient clinic, regular team meetings, or both?
  - *Team Meetings.* It can be a challenge to arrange meetings with all team members on an ad hoc basis. Setting a monthly team meeting time that members can lock into their schedules in advance will help everyone on the team integrate their participation on the team into their regular duties.
  - *Monthly or Quarterly DSD Clinic.* One or more of the divisions represented on the team may already hold a weekly clinic. If half a day can be set aside once a month or once a quarter during clinic time and in that space, the DSD clinic can be held without the need for additional resources.

### 2.4. Interview Team Members

The interviews with team members represent the most revelatory step in the planning process. It is the step that can uncover the core motivations for creating a team, a wealth of ideas for how the team should operate, and the possible barriers that lie between concept and operation. In what follows, we will demonstrate the utility of identifying areas of team agreement, and the necessity of identifying areas where team members' goals and values diverge. Unveiling diverse, previously unarticulated perspectives about how the team should function and specific aspects of care early on will minimize tension, haggling, and ease the resolution of conflicting opinions, thus improving communication with and recommendations to patients and parents.

The team's coordinator (or another member with sufficient time and interest) can be responsible for interviewing each member. It is essential that every team member be interviewed. The professional hierarchies that often infuse hospital dynamics can interfere with team building, and an extra effort should be made to be aware of these tendencies, and to ensure that the voices of all team members are equally valued, solicited, and heard. Team members in some roles may not be able to answer all of the interview questions (e.g., an administrator may not be able to answer questions about a clinical procedure), but everyone should be asked each question, and given the opportunity to give their perspective.

The topics to be covered are discussed in greater detail below, and an example of a sample team member interview protocol can be found in Table 1. Each interview can be expected to take approximately one hour. A robust interview process will include recording and transcribing the interview for accuracy, but if this is prohibitively time-intensive, the interviewer can take detailed notes. Alternatively, team members can be given an electronic copy of the interview questions, and given the option to type responses and submit a hard copy to the coordinator anonymously. There are five topics covered in the interview: (1) departmental procedures and current practices, (2) vision and goals, (3) team functioning, (4) values, and (5) perceptions.

Department procedures and current practices will provide a baseline overview, answering the questions, “where are we now, and what do we have to work with?” This section will capture the organization of divisions represented on the team, how these divisions typically communicate with one another about patients, and what resources are available from each division. The information that is gathered from these interviews will shape how the team will operate, especially when rotations and working hours make it impossible for all of the team members to be on site. The team should contemplate possible scenarios (such as when a child with ambiguous genitalia is born in the hospital at 3 AM) when developing a set of operational guidelines.

The vision and goals section of the interview attempts two things: to get team members to start thinking about their own vision and goals for the team in a concrete and communicable way, and to gather each member's ideas on these topics for comparison. Each team member, whether or not they may be aware of it, has an idea of why he or she wants to be part of the team, why such a team is important, what elements of care can be improved and how to improve them, what the short- and long-term goals of team operation should be, and what outcomes they want to see in patients and patients' families in the future. The vision and goals section includes general, open-ended questions (e.g., “What about the way DSDs are managed now do you hope will be changed by this program?”), and more specific procedural questions (e.g., “When a baby is born with DSD [inpatient], what do you think are the most important things to be done: in the first 24 hours/in the first week/in the first month?”). The information elicited from this section will serve as the basis for the team operation guidelines (TOG), a tool to be described in step 6.

Although team members may be familiar with the concept of teamwork, individual members' ideas of what good teamwork or team functioning entails can vary and, if unaddressed, can hinder team functioning. For example, one member's ideal team governance model could be a communally run and wholly democratic team, with no one voice valued more than another. Another member may value efficiency above all else, and envision an ideal team as having one leader who keeps meetings on track, presents contested issues for a brief debate, then makes unilateral decisions. If both of these people are on the same team, they may each think that they are championing great teamwork, but the work of the team will only be made more difficult as long as the conflicting approaches are unaddressed. Team development research shows that effective teams do have a leader [20–22], but the team will need to determine what style of leadership is most appropriate and agree upon how the team will operate. The team functioning section of the interviews will address this.

Much as each team member has a set of goals and visions for the team, so too does each person hold a set of values relative to how they believe DSD ought to be addressed and what constitutes good care and good outcomes. Ideally, standards of practice will be guided by the best available evidence, but when long-term outcome evidence is lacking, or when available evidence can be interpreted in more than one way, it is important that teams have a framework within which different interpretations, values, and opinions can be debated. The values section of the interview specifically asks where people stand on some potentially controversial issues, such as those pertaining to genital and gonadal surgery, when and how to disclose diagnosis information (to the child, to family members, babysitters, and friends), or in some cases questions of gender assignment. The Consensus Statement, for example, offers specific recommendations pertaining to genital and gonadal surgery [4], and a more recent review article has raised potentially controversial recommendations pertaining to gender assignment [23]. These types of issues can be a source of tremendous difference of opinion and tension among team members, confronting not only long-standing habits of practice, but deeply held beliefs about gender, sexuality, and the role of surgical intervention. One team member may value a normalized genital appearance over future sexual function, another may value preserving sexual function and potential fertility above all else, while another may most value future psychological outcomes and their hypothesized antecedent factors. One team member may believe that gender assignment of children with ambiguous genitalia should be accompanied by cosmetic genital surgery, while another team member may believe that cosmetic genital surgery should be delayed until the child is old enough to take part in the decision making process. In these cases, evidence may not always present a clear path forward, so team members' rationale for decision making—inclusive of values, beliefs, and opinions—should be explored.

It is not necessary for everyone on the team to hold the same values or reconcile differences in beliefs, however, understanding and acknowledging values—and their role in treatment recommendation more generally—is a core tenet of team development and patient care. Eliciting values can be as simple as having team members explain their reasoning behind a recommendation. Given a diagnosis, what does the provider think is the most important outcome to achieve? What benefits and drawbacks do they see as needing to be balanced? The goal is to understand one another's views and agree on a process for how to present a united and well-reasoned recommendation to parents. As the team begins to operate, it is important to keep assessing and discussing team members' values and how they influence treatment recommendations and decisions. Views are likely to change over time, and the team will need to address new evidence as it emerges and make treatment decisions in light of this new evidence.

Finally, the perceptions section of the interview aims to capture any opinions, concerns, and goals that may not have already been addressed. For example, suppose a team member is concerned about the impact that hospital politics or staff hierarchies will have on team functioning. This may not have naturally come up in the team functioning section, but could be a very real issue, and could keep the team from moving forward if left unaddressed.

### 2.5. Analyze Responses

Analyzing interview responses involves identifying the areas in which the team members agree, and the areas in which they show disagreement or a diversity of viewpoints. The analysis need is not to be approached with the systematic rigor of qualitative research analysis, but it should be undertaken by someone with an aptitude for abstract thinking and an ability to recognize and extract themes that arise across interviews. Specifically, the analysis should extract common goals regarding team operation, communication, important elements of patient care, and desired outcomes. The analysis should also identify areas of disagreement, which are opportunities for further team conversation. Common goals will be used in the construction of a logic model (a visual flow chart that illustrates the relationship between program components, activities, and goals, and how they are intended to produce the desired outcomes [24]) and a set of proposed TOG.

Areas in which varying opinions are likely to shape treatment recommendations represent opportunities for further team conversation. These may include differing opinions on the team's referral mechanism, treatment involving genital or gonadal surgery, the degree of importance team members place on psychosocial and peer support, or even what type of care constitutes psychosocial support. Points of variation are of critical importance because they hold the potential either to immobilize the team at the outset if left unacknowledged, or to create a robust foundation for collaboration and success. Articulating and welcoming contrasting viewpoints also helps a team avoid the perils of “group think,” which can occur when individuals attempt to shape their opinions in a way that conforms to what they imagine the consensus of the group to be [25]. Group think discourages some of the core benefits of multidisciplinary teams, such as the promotion of innovative thinking, articulation of the rationale behind recommendations, and questioning habits of practice. Reporting contrasting or diverse viewpoints also provide its own opportunity to frame the diversity of team member perspectives as an asset, rather than an obstacle. Indeed, the presence of different viewpoints is a necessary and healthy component of all teams [26] and is best viewed as an opportunity for team development and growth, rather than an indication that the team is in distress.

Extracting themes can be done in many ways, and analysts should adopt an approach that makes sense to them. One method is to create a spreadsheet with each question listed in the left column and each team members' response listed across in the row. The analyst can read all responses to one question, identify similar responses that come up repeatedly as common goals, and highlight instances of disparate opinion as opportunities for further conversation. Another method is to read through each interview transcript or set of interview notes, synthesize each response into a phrase, sentence, or (for longer responses) list, and then compare these truncated statements, looking for patterns and outliers. It is important to keep in mind, should this step seem daunting, that all report findings will serve as a jumping-off point for the team. If a common goal is identified, and someone disagrees with it, the team will at that point be successfully talking about the issue, which achieves the broader aim. If a more systematic and rigorous method of analysis is desired, there are several instructive books on qualitative research methods that can serve as a helpful resource (e.g., [27]).

### 2.6. Develop Tools and Report Findings

The final step of the planning process is to report findings to the team. The report should begin with an outline of each division represented on the team, including a brief description of how that division is organized (e.g., “Pediatric endocrinology has [#] attending doctors on rotation, working [#] weeks of rotation at a time…”) and what resources that division can offer the team (e.g., “Pediatric urology holds clinic every Tuesday, and one Tuesday morning per month can be dedicated to the DSD clinic.”). This information will have been gathered in the resource assessment and team interviews. The report should then lay out any internal and external funding opportunities discovered during the resource assessment.

A key section of the findings report should include a detailed description of the areas in which team member opinion varied, framed as opportunities for further team conversation, as discussed above. Variations of opinion that were found to be either most common or most widely diverse should be reported topic by topic, including the range of variation and a brief discussion of the possible implications for treatment recommendation.

The remainder of the information gathered in team member interviews can be used to construct three tools: (1) the logic model, (2) proposed team operation guidelines (TOG), and (3) a 3-stage implementation strategy.

**Logic Model**

A logic model is a visual flow chart that illustrates the relationship between program components, activities, and goals, and how they are intended to produce the desired outcomes [24]. (A sample logic model for a hypothetical DSD team is provided in Table 2). This is a simple way to express the resources (inputs), basic activities (outputs), and common short-, medium-, and long-term goals that were discovered in the resource assessment and interview steps. A logic model can provide context, ensuring that everyone involved in the project is able to situate his or her own role within the larger picture. It is also a powerful tool to support program sustainability and team member accountability, to assess program fidelity, and to make program replication possible in the future [24, 28].

**Proposed Team Operation Guidelines**

The proposed TOG chart is a synthesis of ideas proposed by team members regarding how, and when, critical components of care should be provided to both inpatient and outpatient families (a sample set of proposed TOG is provided in Table 3). The guidelines are not rigid recommendations; they should be viewed as a starting point for team review and consideration. To create these guidelines, collect and consolidate team members' thoughts and ideas about how a team should operate (from team member interviews) and synthesize them into a set of proposed operational steps. These operational steps should address procedures for ideal operation, as well as contingency plans for when team members are not on site when a new patient arrives (by birth or as an outpatient). The TOG should include ideas for team operation when a physician who is not on the team comes on as part of the rotation schedule. How will the team operate when a baby with ambiguous genitalia or possible DSD is born outside of the regular working hours of the team coordinator or the assigned first-response psychosocial support provider? Providers involved in DSD care who are not on the team (such as delivery room personnel and medical staff in the pediatric endocrinology and urology divisions) should be incorporated into operation guidelines. When consolidating team member ideas for the TOG, err on the side of inclusivity; even if one person has an idea or specific component that is important to them that the rest of the team may not support, include it. The proposed TOG should be reviewed, revised, and finalized by the team as a group.

**3-stage Implementation Strategy**

The final tool to provide the team is a concrete set of next steps, presented as a 3-stage implementation strategy. The first stage—preparation and piloting—should target the immediate items that need to be accomplished in order for the team to be prepared to operate. Accessing resources and reviewing opportunities for further team conversation and the proposed TOG should be included in the first stage, along with tying up any loose ends that may be preventing the team from taking its first steps forward.

The second stage, establishing full operation, highlights the deliverables associated with taking the team from a pilot project to a fully operational program. This stage should include the remaining logistical or operational goals that the team wants to achieve from a systems perspective, such that by the end of this stage, the members' self-defined criteria for what they want the team to be and achieve are realized.

The last stage, maintenance and sustainability, reflects ongoing operations connected to the team's long-term goals, such as gathering data for research, networking with other DSD teams, long-term followup with patient families over the lifespan, and conducting periodic program evaluations. The content of these stages will be different for every team, depending on what its members envision a fully operational, successful, and mission-driven team to be.

## 3. Conclusion

A gap exists between the need for comprehensive multidisciplinary DSD teams, and the tools provided to those directly involved in DSD care to plan and implement MDTs. As a new era of medical management emerges that demands collaborative and whole-systems treatment for these complex conditions and the families affected by them, the medical community stands in a unique window of opportunity to develop the next standard of DSD care. The process proposed by the above model creates the foundation for long-term success and the achievement of articulated outcomes for patients.

## Figures and Tables

**Table 1:** Sample team member interview.

Current practices	
Please describe your division's organization. How many people are in the division?	
What does rotation and on-call look like? Do you have social workers?	
Psychologists/psychiatrists? Departmental coordinator?	
Does your division collect data about patient outcomes? What kinds of outcome information does your division or	
department collect and track?	
What happens in your division when a baby with DSD is born in the hospital (inpatient)?	
When and how is your division brought into the case?	
What is your division's role in outpatient DSD case management? How does it differ from your role in inpatient case management?	

Vision/goals	

Desired team outcomes.	
What do you view as a desired short-term (0–6 months) outcome of this team? Where do you see the team 6 months from now?	
What do you view as a desired medium-term (6 months–3 years) outcome of this team? Where do you see the team 3 years from now?	
What do you view as a desired long-term outcome of this team? What does a completely formed, fully operational team look like to you?	
Desired patient outcomes.	
What kinds of measurable patient outcomes would you like to see in the short term (within 0–6 months of team implementation)?	
What kinds of measurable patient outcomes would you like to see in the medium term (within 6 months–3 years of team implementation)?	
What do you view as a desired long-term (patient impact) outcome of this team?	
What is it about the way DSDs are managed now that you hope will be changed by this program?	
Why is a program like this important?	
When a baby is born with DSD (inpatient), what do you think are the most important things to be done:	
in the first 24 hours	
in the first week	
in the first month	
When a child with DSD and family first come in (outpatient), what do you think are the most important things to be done:	
in the first 24 hours	
in the first week	
in adolescence (prepuberty)	
in adolescence (postpuberty)	
during transition to adulthood	
What do you want to know from DSD patients (when/if patient feedback is available) and their families who come to this hospital about their experience with the team?	
How do you think we should gather this data?	
Should this data be used strictly internally, or available for future research purposes?	
How do you envision this team operating? For inpatient and outpatient cases, start from the beginning. How should the team be notified? Who does what, and when?	
How should we incorporate other providers who are not on the team, but who will be involved in DSD care (such as staff in the delivery room, or attending physicians if a team member is not on site)?	

Team functioning	

What are the essential ingredients to good team functioning, in your opinion?	
What are the essential steps towards forming a functional team, in your opinion?	
How often, and under what circumstances, do you envision the team meeting?	
Do you envision this team having a leader?	
Do you have any specific concerns about, or hopes for, this team's dynamic?	

Values	

Disclosure of information to patients.	
Do you think that considering disclosure is an important component of care?	
What information do you think should be disclosed to patients?	
When do you think this information should be disclosed?	
How do you think this information should be disclosed?	
What parents should know:	
What do you think is important for parents to know in order for them to make decisions about their child?	
The following are some core concepts of care recommended by the Consensus Statement. Are you comfortable with these concepts of care? Do any of these recommendations seem inappropriate to you? Are there any practice points that you think are missing?	
All individuals should receive gender assignment.	
Gender assignment should be avoided until a comprehensive evaluation is completed.	
Open communication with families is essential and participation in decision making is encouraged.	
Emphasis of surgical intervention should be on functional outcome rather than strictly on cosmetic appearance.	
Feminizing surgery should only be considered in cases of severe virilization.	
Surgical management of DSD should consider options that will maximize the chances of fertility.	
The streak gonad in a patient with MGD-raised male should be removed laparoscopically (or by laparotomy) in early childhood. Bilateral gonadectomy is performed in early childhood in females (bilateral streak gonads) with gonadal dysgenesis and	
Y-chromosome material. In patients with androgen biosynthetic defects raised female, gonadectomy should be performed before puberty. A scrotal testis in patients with gonadal dysgenesis is at risk for malignancy [4, page e492].	
The process of disclosure concerning facts about karyotype, gonadal status, and prospects for future fertility should be a collaborative ongoing action, which requires a flexible, individual-based approach, and should be planned with the parents	
from the time of diagnosis.	
The following are some key components of the model for shared decision making [10]. Are you comfortable with these concepts?	
Establish preferences for information and roles in decision making.	
Perceive and address parents' emotions.	
Define concerns and values.	
Identify options and present evidence.	
Explore parents' ideas and assumptions, correct misperceptions.	
Ensure parental understanding.	
Share responsibility for making a decision.	

Perceptions	

What do you view to be the strengths of, and assets to, this team?	
What do you view to be the potential challenges to team formation and operation?	
What is your main reason for participation on this team?	
What do you care about most when it comes to care for these families?	

**Table 2:** Sample logic model.

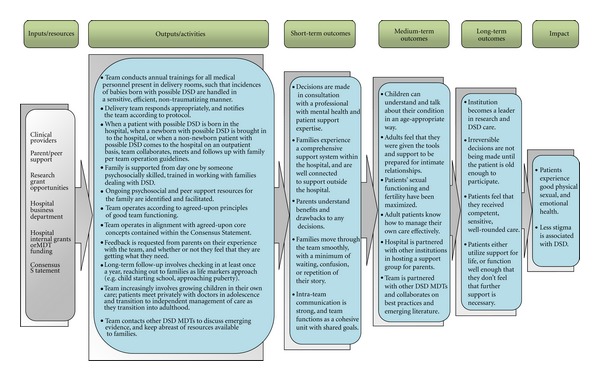

**Table 3:** Sample team operation guidelines.